# A case report of T-box 1 mutation causing phenotypic features of chromosome 22q11.2 deletion syndrome

**DOI:** 10.1186/s40842-019-0087-6

**Published:** 2019-08-13

**Authors:** Raad A. Haddad, Gregory A. Clines, Jennifer A. Wyckoff

**Affiliations:** Division of Metabolism, Endocrinology, and Diabetes (MEND), Department of Internal Medicine, University of Michigan, 24 Frank Lloyd Wright, G-1500, Ann Arbor, MI 48106 USA

**Keywords:** 22q11.2 deletion, DiGeorge syndrome, Hypoparathyroidism, Hypocalcemia

## Abstract

**Background:**

The heterozygous microdeletion of chromosome 22q11.2 results in a spectrum of disorders, including DiGeorge syndrome (DGS) and velocardiofacial syndrome (VCFS), with phenotypic features that can include the classic triad of congenital heart disease (CHD), thymic aplasia and hypoparathyroidism. Such microdeletions are usually detectable by fluorescence in situ hybridization (FISH).

**Case presentation:**

We report a case of a twenty-three year-old female who presented with clinical features of chromosome 22q11.2 deletion syndrome including cardiac anomalies, hypoparathyroidism and dysmorphic facial features. FISH did not reveal a 22q11.2 microdeletion. Further genetic analysis showed T box-1 (TBX1) heterozygous mutation.

**Conclusions:**

The TBX1 gene plays a significant role in the development of fourth pharyngeal arch structures. Mutations of TBX1, which is found at chromosome 22q11.21 can be responsible for the development of syndromes classically associated with chromosome 22q11.2 deletions. This case emphasizes that the TBX1 gene, among other genes, can be responsible for the developmental anomalies seen in these syndromes.

## Background

A wide spectrum of phenotypic features may present as a result of the deletion of chromosome 22q11.2. DiGeorge syndrome (DGS), velocardiofacial syndrome (VCFS), and various other syndromes have been described in association with the deletion involving human chromosome 22q11.2 [1]. The T-box 1 (TBX1) gene, located on chromosome 22q11.21, has a significant role in the differentiation and development of the third and fourth pharyngeal arches into the pharyngeal arch arteries, cardiac outflow tract, thymus, parathyroid glands, and craniofacial structures [2–4]. We report a case of a twenty-three year-old female who presented with phenotypic features of 22q11.2 deletion and was found to have heterozygous mutation of the TBX1 gene.

## Case presentation

**Fig. 1:**
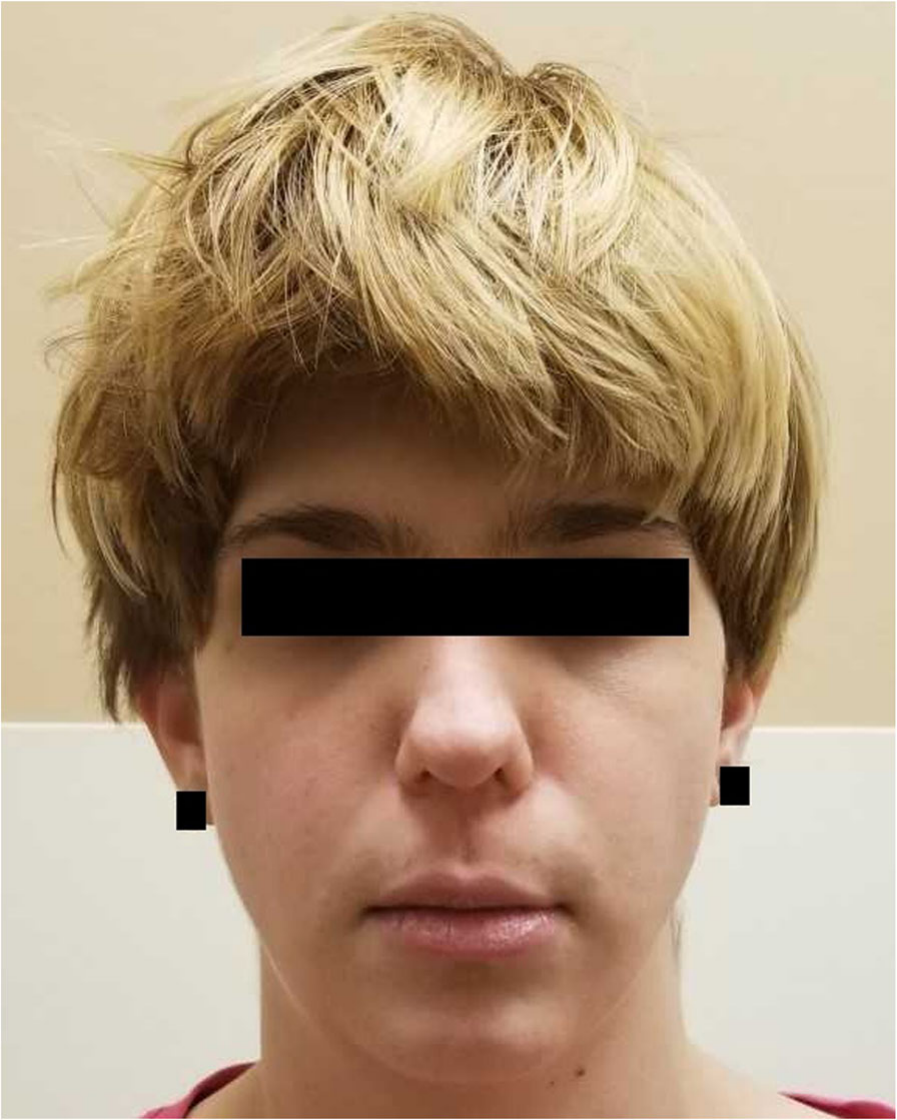
Facial features of the reported case showing dysmorphism of the nose, flattened philtrum and micrognathia

A twenty three year-old female presented to transition her care from pediatric to adult endocrinology. At the age of one year, she had presented to her local emergency department with seizures and was found to have serum calcium of 6.7 mg/dL (reference range 8.2–10.2 mg/dL). Her serum phosphorus level at that time was 7.8 mg/dL (reference range 2.3–4.7 mg/dL) and magnesium of 2.3 mEq/L (reference range 1.5–2.5 mEq/L). Testing performed at that time included an intact parathyroid hormone (PTH) level of 15 pg/mL (reference range 10–65 pg/mL), a 25-hydroxyvitamin D level of 61 ng/mL (reference range of 30–80 ng/mL) and a 1,25-dihydroxyvitamin D level of 45 pg/mL (reference range 15–65 pg/mL), consistent with hypocalcemia due to hypoparathyroidism. There was no history of candidiasis. By two years old, she was also diagnosed with multiple other conditions including hydrocephalus, atrial septal defect, bicuspid aortic valve, left kidney agenesis and thoracolumbar scoliosis. She was later diagnosed with learning disabilities including cognitive and attention deficits, no other psychiatric disorders were diagnosed. Her facial features are shown in Fig. 1 and are noticeable for dysmorphism of the nose, flattened philtrum, micrognathia and hypertelorism (not shown in the figure). She did not have palatal or teeth abnormalities. She underwent an immunological evaluation that was unremarkable. Her biochemical thyroid evaluation was also normal. Her hypoparathyroidism has been managed with calcitriol and calcium supplementation. Her corrected calcium levels were maintained in the range of 8.0–9.5 mg/dL. Her last 24-h urinary calcium was 163.5 mg/dL. She has not developed nephrolithiasis, and renal ultrasound is significant only for an absent left kidney. Given the clinical features that were compatible with 22q11.2 deletion, she initially underwent FISH that did not reveal the targeted deletion. She then had bi-directional DNA sequencing that detected a heterozygous mutation (c.1055 C > T; p.Pro352Leu) resulting in a proline to leucine substitution at amino acid 352 in the TBX1 gene.

## Discussion and conclusions

Chromosome 22q11.2 deletion syndrome accounts for the most common microdeletion disorder in humans with an estimated incidence of 1 in 4000–10,000 live births. [5, 6]. The clinical features of this microdeletion were first described as a triad of congenital heart disease (CHD), thymic aplasia and hypoparathyroidism by Dr. Angelo DiGeorge in 1965. [7, 8]. Other conditions were subsequently described with overlapping phenotypic features including velocardiofacial syndrome (VCFS), conotruncal anomaly face syndrome, and Caylercardiofacial syndrome. [9–11]. It was later concluded that all of these syndromes share the same causative mutation with heterogeneous clinical presentations.

The majority of patients have a 3 Mega-base (Mb) heterozygous deletion of 22q11.2 segment that contains approximately 30 genes responsible for the early development of the pharyngeal arch derivatives, leading to compromised maturity of the craniofacial structures, the upper thorax, the thymus, the parathyroid glands, the heart, and the cardiac outflow tract [12–14]. A wide spectrum of clinical manifestations is observed including cardiac anomalies, immunodeficiency due to thymic aplasia, hypocalcemia due to hypoparathyroidism and dysmorphic facial features. Other findings include palatal, musculoskeletal, gastrointestinal, renal, behavioral, psychiatric (including anxiety and schizophrenia) and cognitive abnormalities (including learning disabilities, attention-deficit, and mental retardation) were also found to be related [15–17]. The clinical presentation of the patient reported here was compatible with the phenotypic features described. Her FISH analysis did not detect the expected deletion. Classically, FISH has been the routine diagnostic test to reveal such microdeletion. However, the reported sensitivity of this test was nearly 78% by Michaelovsky et al. [18]. Routine FISH technique uses a probe mapping to the LCR22A–LCR22B region, thus, deletions outside this region or deletions that are too small to detect can be left unrevealed [8, 19]. In the case mentioned here, further genetic analysis revealed heterozygous mutation of the TBX1 gene (P352L variant). This variant has not been reported before in this syndrome, however, one missense mutation in a nearby residue (G350D) has been reported in association with cardiovascular defects [20].

The TBX1 gene has been extensively studied in mice; heterozygous TBX1 deleted mice were found to have aortic arch defects which represents one of the major characteristics of the 22q11.2 deletion syndrome [21]. Homozygous deletion of this gene in mice led to a wider spectrum of phenotypic characteristics including thymus and parathyroid abnormalities, cardiac outflow tract anomalies, and craniofacial defects [22, 23]. In human studies, Yagi et al. performed mutational analysis in patients with 22q11.2 deletion syndrome features but without the chromosomal deletion; TBX1 heterozygous mutation was found responsible for major phenotypic features in this syndrome, predominantly, cardiac and cardiac outflow tract anomalies [2, 24, 25]. These findings confirmed that TBX1 gene haploinsufficiency plays a crucial role in the development of the 22q11.2 deletion syndromes.

In such cases, a multidisciplinary approach for management is required and depends on the age of presentation and the spectrum of consequences. Patients with this syndrome need individualized and complex medical care with vigilant recognition of the acute and long-term manifestations. From an endocrinology point of view, maintaining calcium homeostasis remains essential to prevent hypocalcaemia. The lack of PTH results in the under conversion of vitamin D (25-hydroxyvitamin D) to its active form (1,25-dihydroxyvitamin D), and thus, low calcium absorption. Supplementation with calcium and active vitamin D (i.e. calcitriol) is the cornerstone of management [26, 27]. These patients also lack the effect of the PTH on renal calcium reabsorption. It is therefore preferred to maintain serum calcium level in the low normal range to prevent hypercalciuria and the subsequent development of nephrolithiasis and renal failure [28]. Some patients may require thiazide diuretics to decrease urinary calcium excretion [29]. In patients who fail to maintain stable serum and urinary calcium on calcium and vitamin D supplementation, recombinant PTH can be given as a second line therapy. Recombinant human PTH (1–84), the native form of PTH, was approved by the U.S. food and drug administration (FDA) in 2015 for use in patients with chronic hypoparathyroidism. After double-blinded, randomized clinical trial reported a reduction of, or even independence from, supplemental calcium and calcitriol with stable serum and urinary calcium levels over 24 weeks [30]. Synthetic PTH (1–34), which is the active form of PTH and in fact FDA approved for use in osteoporosis, also showed favorable effect with improvement of hypercalciuria when compared to conventional therapy with calcitriol and calcium [31, 32].

Over the last two decades, studies have revealed the consequences of TBX1 haploinsufficiency in mice, mimicking the phenotypic features seen in 22q11.2 deletion syndrome. Human studies to date are limited giving the rarity of such mutations. The case reported here replicates the findings that were observed in previous human studies and underscores the fact that TBX1 gene mutation plays a key role in the development of this syndrome.

## Data Availability

The data used in this case report are available in the patient’s medical recordand can be disclosed by the corresponding author on reasonable request.

## Abbreviations

CHD: Congenital heart disease
DGS: DiGeorge syndrome
FISH: Fluorescence in situ hybridization
Mb: Mega-base
PTH: Parathyroid hormone
TBX1: T box-1
VCFS: Velocardiofacial syndrome
